# Incorporation of In Situ Synthesized Nano-Copper Modified Phenol-Formaldehyde Resin to Improve the Mechanical Properties of Chinese Fir: A Preliminary Study

**DOI:** 10.3390/polym13060876

**Published:** 2021-03-12

**Authors:** Fan Li, Cuiyin Ye, Yanhui Huang, Xianmiao Liu, Benhua Fei

**Affiliations:** 1 College of Materials Science and Technology, Beijing Forestry University, Beijing 100083, China; lifanbjfu@163.com (F.L.); phyllisyip@163.com (C.Y.); 2 International Center for Bamboo and Rattan, Beijing 100102, China

**Keywords:** cell walls, micromechanical properties, macromechanical properties, PF resin, modification

## Abstract

Phenol-formaldehyde (PF) resin, modified using nano-copper with varying contents (0 wt%, 1 wt%, 3 wt%), was manufactured to improve the mechanical properties of Chinese fir. The morphology, chemical, micromechanical and micromechanical properties of the samples were determined by transmission electron microscopy (TEM), atomic force microscopy (AFM), environmental scanning electron microscopy (ESEM), Fourier transform infrared spectroscopy (FTIR), nanoindentation (NI) and traditional mechanical testing. The TEM and AFM results indicated that the in situ synthesized nano-copper particles were well-dispersed, and spherical, with a diameter of about 70 nm in PF resin. From the FTIR chemical changes detected by FTIR inferred that the nano-copper modified PF resin penetrated into the Chinese fir cell walls and interacted with the acetyl groups of hemicellulose by forming a crosslinked structure. Accordingly, the micro-mechanical properties of the Chinese fir cell walls were enhanced after treatment with nano-copper modified PF resin. The filling of the PF-1-Cu resin (1 wt% nano-copper) in the wood resulted in 13.7% and 22.2% increases in the elastic modulus (MOE) and hardness, respectively, of the cell walls. Besides, the impact toughness and compressive strength of the Chinese fir impregnated with PF-1-Cu resin were 21.8% and 8.2% higher than that of the PF-0-Cu resin. Therefore, in situ synthesized nano-copper-modified PF resin is a powerful treatment method for Chinese fir due to improved diffusive properties and reinforcement of the mechanical properties.

## 1. Introduction

Chinese fir is the main planted timber species in southern China. Wood from Chinese fir forests has become an important industrial raw material for broad commercial use, because of its aesthetic, good adaptability to the environment, and shorter growth cycle. However, Chinese fir presents several major disadvantages, such as low surface hardness and dimensional stability, which limit its commercial and practical applications [1].

To improve the surface hardness and stability of plantation wood, various modification methods have been developed, including chemical impregnation, densification, thermal treatment, and surface coating [2,3,4,5]. Chemical impregnation has been a powerful method to improve the mechanical properties of wood and promote its industrial utilization [6,7,8,9].

Low molecular weight phenolic resin as a common impregnation modifier has been widely used to improve wood properties [8,10,11,12]. Among the modifiers, water-soluble low molecular weight phenol-formaldehyde (PF) resin is known for its low initial viscosity, low-cost, low-toxicity, and non-flammability; they can also easily diffuse into wood cell walls, and enhance the properties of wood [13]. PF resin can also form a complex, cross-linked structure with components in its wood cell walls [14,15,16]. Other studies have shown that water-soluble PF resin can significantly improve the dimensional stability, surface hardness and compressive properties of wood [14,15,16,17,18].

Phenolic resin as an impregnation modifier has numerous advantages. But limited by its chemical structure, PF resin is still characterized by several deficiencies. The phenolic hydroxyl group and methylene of PF resin are easily oxidized, which can reduce its oxidative resistance [18]. The cured PF, which is only connected by methylene, results in the high density of the rigid groups (benzene ring). Notably, large steric hindrance and small rotational degrees of freedom enhance the brittleness of pure PF. Accordingly, brittleness increases when PF resin cures on the wood surface, which significantly limits its application range and service life. Therefore, PF resin must be modified. Nanoparticles such as TiO_2_, Al_2_O_3_, CuO, ZnO have been widely used in recent years to enhance the physical and mechanical properties of PF resin [19,20,21,22]. It has been reported that the inherent brittleness of PF resin being used as a friction material (plastic field) is one of its limiting conditions, whereas nano copper has excellent properties which can be used to enhance the strength, hardness, toughness and chemical resistance of PF resin. [23,24]. Nano-copper can also reduce the apparent activation energy and curing time of PF resins [25].

Unfortunately, nanoparticles-reinforced PF resin generally suffers from some problems. Generally, nanoparticles can easily agglomerate in the PF resin matrix and the PF resin exhibits weak interfacial bonding with reinforced nanoparticles. Therefore, the mechanical properties of PF resin were reduced. These factors limit the application of nanomaterials in PF resin modification. In addition, in the wood modification, nano-copper and PF resin are typically used separately to modify wood to improve their corrosion resistance, and mechanical properties, respectively [26,27]. To the best of our knowledge, researchers rarely combine nano-copper with PF resin to modify wood, because of the large aggregations of nano-copper and very limited studies focusing on the mechanical properties of wood cell walls after impregnation with modified PF resin.

Therefore, to address the problem of nano-copper agglomeration, many attempts have been made to explore effective methods for dispersing nanoparticles in PF resins. To render nano-copper more uniformly dispersed in PF resins, polyvinyl pyrrolidone (PVP) is used as the surface stabilizer, reducing agent and dispersant. The bonding strength of the filler-matrix is thus improved. Subsequently, a nano-copper modified PF resin with good dispersibility was synthesized in situ, alleviating the brittleness of wood treated with PF resin. The morphology, chemical, micromechanical and macromechanical properties of the treated and control samples were determined by transmission electron microscopy (TEM), transmission electron microscopy (AFM), environmental scanning electron microscopy (ESEM), Fourier transform infrared spectroscopy (FTIR) and nanoindentation (NI), etc., to investigate the interaction mechanism of the modified resin with wood. This study can provide an effective method for the preparation of high-quality wood products, such as high mechanical properties and dimensional instability, used for outdoor or high humidity environments.

## 2. Materials and Methods

### 2.1. Materials

**Figure 1:**
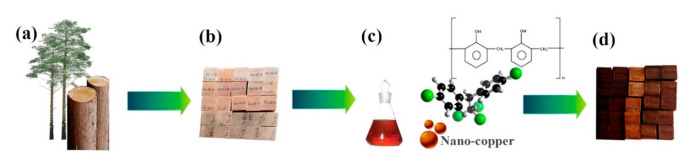
Schematic illustration of the sample preparation. (**a**) Chinese fir logs, (**b**) Sawed samples, (**c**) Preparation of nano-copper modified PF resin, (**d**) Chinese fir samples impregnated with resin.

In this study, 40-year-old Chinese fir (*Cunninghamia lanceolata*) was sourced from the Gongyi Forest Farm in the Huangshan, Anhui Province of China. Samples were chosen at a trunk height of 1.5 m. The specimens were cut into a final size of 10 × 5 × 5 mm^3^ in the longitudinal (L), tangential (T), and radial (R) directions. They were then numbered by the sawing sequence from the initial location (Figure 1). Among them, odd samples were modified with vacuum and high-pressure impregnation treatment; even samples were marked as control samples. All reagents were purchased from Beijing Chemicals in Beijing, China and used as received.

### 2.2. Preparation of PF Resin

The modified PF resins were synthesized in a three-necked flask. Phenol and formalin were first fed into the flask. The reaction of phenol with formaldehyde proceeded at 70–80 °C under stirring for 1 h in the reactor and was catalyzed by NaOH (40 wt% dissolved in water). Then, it was rapidly stopped by placing the reactor in a cold-water bath. Subsequently, the formaldehyde added into the reactor was vigorously stirred at 75 °C for 2 h. After that, it was quickly cooled to 40 °C (the molar ratio of formaldehyde, phenol and NaOH was 1:2.1:0.2).

### 2.3. Preparation of Nano-Copper Modified PF Resin

The percentage of nano-copper in PF (by weight) was controlled by changing the amount of CuSO_4_ and prepolymer to obtain small particle sizes and good dispersion, and PVP was also mixed the polymer to avoid of nano-copper agglomeration during synthesis. Four groups of samples were prepared: the control group and the groups with 0%, 1%, and 3% nano-copper-modified PF (Table 1).

Preparation of the PF-1-Cu resin: In the fabrication of PF-1-Cu, the former part was prepared using the same method as that of PF resin. The amount of composition added during the experiment is listed in Table 1. Until the PF resin in the three-necked flask was quickly cooled below 60 ℃, the mixture (sodium pyrophosphate and potassium sodium tartrate was dissolved in the aqueous solution of formaldehyde) was added. Under vigorous stirring, the 40% NaOH was added for 20 min to adjust the pH to 12. Subsequently, a solution of copper sulfate (CuSO_4_, disodium ethylenediamine tetraacetate (EDTA-2Na), and PVP dissolved in H_2_O, with pH adjusted to 11.5 by using NaOH (40%)) was added into the three-necked flask. It was stirred at 50 ± 5 °C for 30 min and NaOH (40%) was added to maintain the pH at 12 ± 0.5. The temperature was set to 70 ± 5 °C for another period of 1 h, and then cooled to 40 °C to obtain the PF-1-Cu resin synthesized in situ. Similarly, the PF-3-Cu resin was to be synthesized in accordance with Table 1. The viscosities and solid contents of the modified PF resins are listed in Table 2.

Cu^2+^ + 4OH^−^ + 2HCHO = Cu + 2HCOO^−^ + H_2_ + 2H_2_O (1)

### 2.4. Vacuum and High-Pressure Impregnation Treatment

Before impregnation, the moisture contents (MC) of all samples were conditioned to 12% at 65% relative humidity (RH) and 20 °C. The samples were impregnated with in situ synthesized nano-copper modified PF resin in a vacuum from different groups under pressurized conditions. First, the resin was diluted with deionized water to a concentration of 25%. Then, the samples were impregnated in the resin and placed in a vacuum for 2–3 h to ensure the resin had been adsorbed. Next, the process was conducted at 0.5 MPa for 1 h, then at 0.6 MPa for 2 h, and finally at 0.7 MPa for 4 h. The samples were then placed in an oven, dried at 45 °C for 4 h, 70 °C for 6 h, and 103 °C for gradual drying in the end.

### 2.5. Characterization

Particle sizes and morphologies were characterized by TEM (H-800, Hitachi Inc., Tokyo, Japan). The resin was dropped on the copper grid, and after drying, the morphological characteristics of the copper particles were observed by TEM. The acceleration voltage was 80 kV, and the magnification level was 40–200 Kx.

The morphological and chemical compositions of impregnated Chinese fir were investigated by environmental scanning electron microscope coupled with an energy dispersive spectrometer (ESEM–EDS) (SU8010, Hitachi Inc., Tokyo, Japan). The samples were cut into 0.15 × 5 × 5 mm^3^ (L × R × T) pieces with a microtome. After the surface was sprayed with gold, the samples were observed at an operating voltage of 15 kV.

The sample surfaces of the nano-copper particles were performed with an AFM (Multimode 8, Bruker Inc., Santa Barbara, CA, USA) under PF-QNM imaging mode. The surface topography of the PF-1-Cu was recorded over an area of 1 × 1 μm^2^ in tapping mode, at a high resolution with a sharp silicon tip (0.5 N/m). The two-dimensional (2D) and three-dimensional (3D) images were obtained at the room temperature (24 ± 1 °C).

Chemical changes in the control and nano-copper modified Chinese fir samples were characterized using a Nicolet IN10 Fourier infrared spectrometer (Thermo Scientific, Waltham, Waltham, MA, USA) within the range of 4000–800 cm^−1^, using 64 scans with a resolution of 4 cm^−1^. Thereafter, the FTIR spectra were processed using Origin 9.1 software (OriginLab Inc., Northampton, Waltham, MA, USA). The corrected spectra were subjected to three corrections, namely, atmospheric, flat, and baseline offset corrections.

The mechanical properties of the wood are the main indicators of resistance to external forces during wood application. Among these factors, impact toughness and compressive strength are one of the most important factors. The impact strength of specimens measuring 300 × 20 × 20 mm^3^ was determined using a pendulum impact tester (Tinius Olsen Inc., Horsham, PA, USA) according to ISO 13061-10. The compressive strength parallel to the grain was measured on a CMT 5205 test machine (MTS Systems Co. Ltd., Shenzhen, Guangdong, China), the sample 20 × 20 × 30 mm^3^ according to ISO 3129:2012.

Besides, to evaluate the effects of nano-copper-modified low molecular weight PF on the nanomechanical properties of the wood cell walls, an NI (Triboindenter, Hysitron Inc., Minneapolis, MN, USA) with an in situ imaging function was used. The samples were glued to a sample holder and flattened with a diamond knife. The samples were then conditioned in the nanoindentation test chamber for more than 48 h. Subsequently, the test was performed using a Berkovich diamond indenter (the tip radius of under 100 nm), loaded in 5 s to a peak force of 250 μN, with the maximum force held for 6 s, and ultimately unloaded in 5 s. The NI hardness and modulus of elasticity (MOE) of the samples were calculated using the method described in the literature (Yu et al., 2007; Huang et al., 2013).

High-resolution electrospray ionization mass spectrometry of PF-1-Cu samples was acquired using a Bruker Apex IV FTMS (Bruker Inc., Bremen, Germany). It was shown that the modified resin mostly varied from 343 to 615 g/mol. This means that the modified resin exhibited low molecular weight, which aided in better penetration into the wood cell walls.

## 3. Results and Discussion

### 3.1. Dispersion of Low Molecular Weight PF and Nano-Copper in the Wood Cell Walls

**Figure 2:**
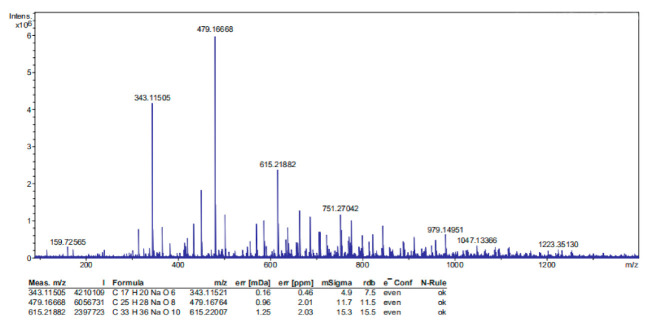
High resolution mass spectrum of the molecular weight distribution of 1 wt% nano-copper modified low molecular weight PF resin.

The penetration depth of modified PF resin into wood cell walls was generally affected t by the molecular weight of the PF resin oligomers and the dispersibility of nano particles. Figure 2 shows that the molecular weight of the PF resin is relatively low (mostly less than 500 g/mol). In addition, to elucidate the permeability of the nano-copper modified low molecular weight PF resin in the wood cell walls, it is essential to study the distribution and diameter of the in situ synthesized nano-copper particles.

**Figure 3:**
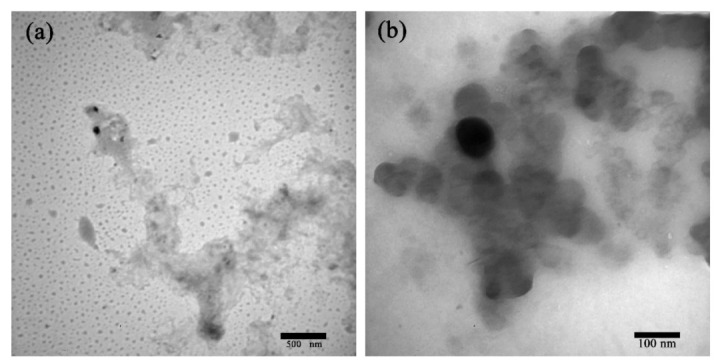
TEM image of the 1 wt% nano-copper modified low molecular weight PF resin when the scale bars are (**a**) 500 nm and (**b**) 100 nm, respectively.

To further verify the more detailed nano-copper modified PF resin microstructure, Figure 3 shows the TEM image of the modified PF resin with 1 wt% in situ synthesized nano-copper particles. The black particles in the figure represent the in situ synthesized nano-copper, whereas the gray portion denotes the low molecular weight PF resin. As indicated in the figure, the nano-copper is well-dispersed throughout the PF resin. Distinct from that of the PF resin micrograph, the morphology of the nano-copper particles could be easily identified with spherical in geometry, which agrees with previous reports [28]. The diameters of the nano-copper particles were in the nanometer range and their distribution showed no agglomeration.

**Figure 4:**
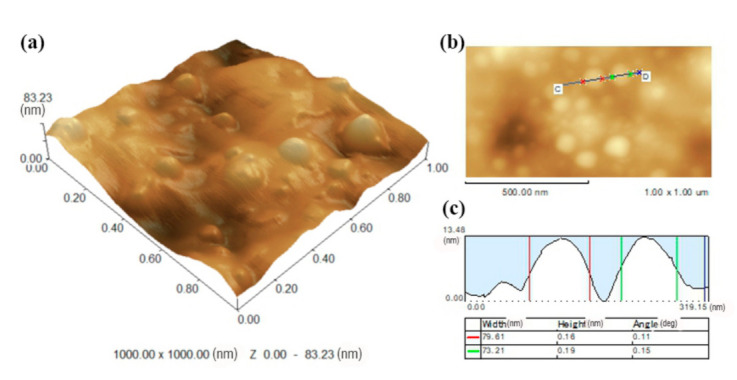
AFM imaging of 1 wt% nano-copper-modified PF resin. (**a**) and (**b**) are the three-dimensional and two-dimensional image of AFM, respectively; (**c**) the section analysis image of AFM.

The AFM image not only clearly reveals the distribution of nano-copper in the PF resin, but also obtains the diameter of the nano-copper accurately and concretely. Figure 4 is the AFM image of 1 wt% nano-copper modified PF resin, which shows that the nanoparticles were dispersed well throughout the sample, with average diameters of about 70 nm. This result was attributable to the use of in situ synthesis method to prepare the nano-copper modified PF resin.

Nanoparticles exhibit high surface activity that can easily aggregate. However, the nano-copper in this experiment did not aggregate for the following reasons: (1) As the polycondensation reaction was initiated, and nanosized copper particles suffer from steric hindrance ascribed to the increasing molecular weight and viscosity of the PF resin prepolymer [29]). (2) The PVP added to the reaction system acted as a protective layer by coordinating atomic N, O, and Cu. Simultaneously, the long chains of aliphatic C-H extended to the surrounding environment and form a stereoscopic barrier to prevent the agglomeration of copper particles. PVP was used as a dispersant and stabilizer in the process of nano-copper modified PF resin, and the stable PVP-coated Cu nanoparticles were prepared using a simple chemical route to prevent the agglomeration of copper particles [30]. Copper ions were reduced in the presence of PVP.

**Figure 5:**
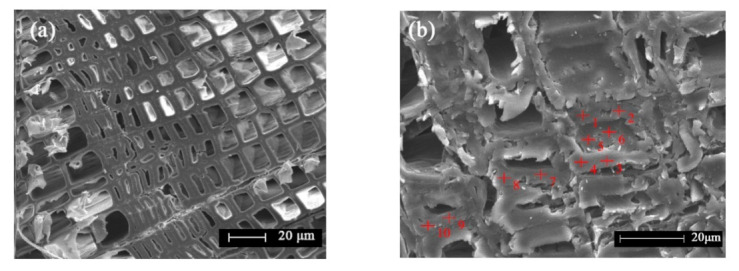
ESEM image of the control samples (**a**) and PF-1-Cu samples (**b**).

The improvement of the nano-copper-modified PF resin on the properties of Chinese fir mainly depends on the permeability and uniformity distribution of nano-copper and the resin in the cell walls. After impregnating PF resin with nano-copper content of 0 wt%, 1 wt%, and 3 wt%, the weight gain rates of Chinese fir were 26.67%, 19.36%, and 18.75%, respectively. The morphology of the treated and control samples were observed directly by ESEM (Figure 5). It is clear that nano-copper-modified PF resin entered the cell lumens of the Chinese fir. Moreover, the modified PF molecules freely diffused into the intercellular spaces of the wood under vacuum and pressure conditions. In addition, it was also observed that no obvious boundary was found between the cell walls and the filled modified PF resin, which may indicate that some PF resin has penetrated into the S2 layers of the cell walls and may interact with one another.

Table 3 shows the copper containing percentage of the 10 points taken in Figure 5. The calculation is based on the weights of C, O, and Cu in the EDS test results.

The ESEM test was used to study the permeability of nano-copper modified PF in Chinese fir, and the results are listed in Table 3. Among them, points 5 and 6 were located in the cell lumen, while the remaining points are located on the cell walls. Table 3 shows that the nano-copper modified low molecular weight PF was diffused and penetrated widely not only into the cell lumens but also into the cell walls. The relative percentage of nano-copper was high in the PF-1-Cu samples. The average percentage of nano-copper in the cell lumens was 2.13%, which was nearly 24% higher than that in the wood cell walls (1.72%). This result indicated that the modified PF resin was easier to penetrate into the cell lumens than the cell walls. Smith et al. (1985) and Imamura et al. (1998) reported that the molecular weight of a resin was crucial to its penetration into wood cell walls and revealed that the presence of resin in the cell walls than in the cell lumens can alter the properties of wood to greater extents [31,32]. Therefore, the penetration of the modified PF resin is highly influential on the mechanical properties of cell walls.

### 3.2. Improvement of the Micromechanical Properties of the Wood Cell Walls

**Figure 6:**
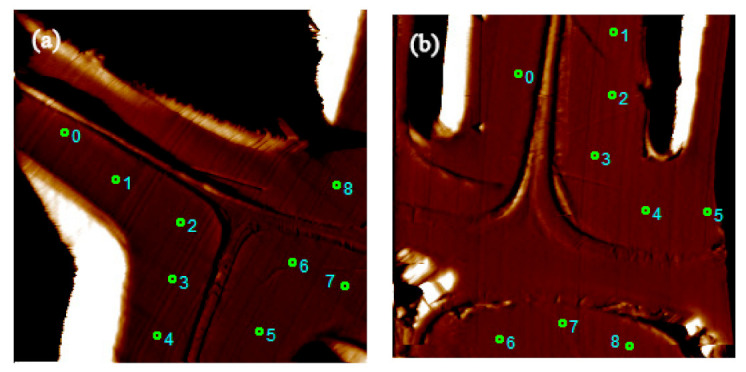
Nanoindentation images of Chinese fir marked with measurement location. (**a**) control sample, (**b**) PF-1-Cu sample.

**Figure 7:**
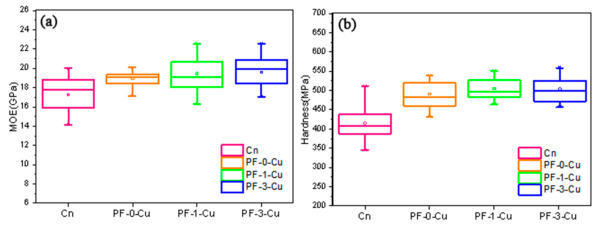
Micromechanical properties of samples before and after modification treatment (**a**) elastic modulus, (**b**) hardness.

Figure 6 shows the images of nanoindentations, showing the measurement locations of untreated and PF-1-Cu-treated Chinese fir, respectively, to evaluate the effects of nano-copper modified PF resin on the NI MOE and hardness of the wood cell walls. Figure 7 and Table 4 display the changes in the mechanical properties of the cell walls before and after modification.

The variation coefficient of NI MOE of the samples was small, less than 10% for each sample. The average NI MOE and hardness values of the cell walls in the samples are listed in Table 4. As shown in the table, the average NI MOE value of the untreated Chinese fir was 17.3 GPa, and that of the sample impregnated with PF-0-Cu resin was 18.9 GPa, which was 9.9% higher than that of the former. Obviously, the average NI MOE of the PF-3-Cu samples was higher (maximum of 19.6 GPa), which was 13.7% higher than the control Chinese fir. However, the NI MOE values of PF-1-Cu and PF-3-Cu samples were similar.

The NI hardness value of the control samples was 415.1 MPa, and that of the PF-0-Cu samples reached 491.4 MPa, reflecting a difference of approximately 18.4%. After 1 wt% nano-copper was added, the NI hardness of the samples was as high as 507.3 MPa, which was 22.2% higher than the control samples. The results prove that a 1% addition of nano-copper could improve the mechanical properties of the wood cell walls. Furthermore, the variation coefficient of NI hardness with different contents of nano-copper was within a small range.

According to the literature, it can be inferred that the modified PF resin diffused into the nanopores of the wood cell walls and form crosslinked networks, improving the mechanical properties of wood [33]. In the current experiment, the mechanical properties of the wood cell walls were improved through physical and chemical modification. It was further confirmed that nano-copper-modified low molecular weight PF could be evenly distributed in the cell walls and enhanced the mechanical properties of the cell walls. Furthermore, the increase in NI hardness was significantly more than the increase of NI MOE, which was attributed to the lower MOE and higher hardness of the modified PF resin polymers [34]. In addition, the average values of the NI MOE and hardness values of PF-1-Cu and PF-3-Cu samples were similar, which is likely, because the PF resin with 1 wt% nano-copper to modified Chinese fir is better than the latter. The reason was that the low content of nano-copper not only economically saves materials, but also prevents the agglomeration of nano-copper.

### 3.3. Mechanical Properties of Chinese Fir after Impregnation

**Figure 8:**
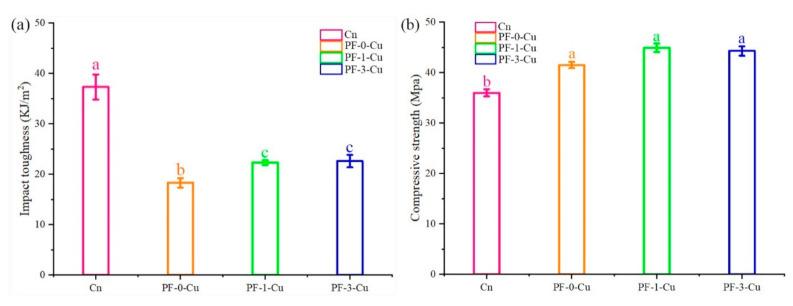
Mechanical properties of the control, PF-0-Cu, PF-1-Cu, and PF-3-Cu samples. (**a**) impact toughness, (**b**) compressive strength.

Relative to that of samples treated using PF-0-Cu resin, the impact toughness of the Chinese fir impregnated by PF-1-Cu resin increased by 21.8%, while that of the PF-3-Cu resin decreased by 1.4% (Figure 8a). The results indicated a significant decrease in the brittleness of the samples impregnated with in situ synthesized nano-copper modified PF resin. The PF-1-Cu resin absorbed more impact force and increased the toughness of the Chinese fir [35,36,37].

Compression experiments parallel to the growth direction were performed, shown in Figure 8b. The Chinese fir impregnated with PF-0-Cu showed compressive strength was 15.3% higher than the control samples. However, compressive strength of the wood treated with PF-1-Cu resin significantly improved by 24.7% relative to that of the untreated wood, making this material possible for commercial applications. This may be due to the fact that nano-copper well-dispersed in PF played an indispensable role in improving the compressive strength of Chinese fir.

### 3.4. FTIR Analysis of the Combination of Low Molecular Weight PF, Nano-Copper and Wood Cell Walls

**Figure 9:**
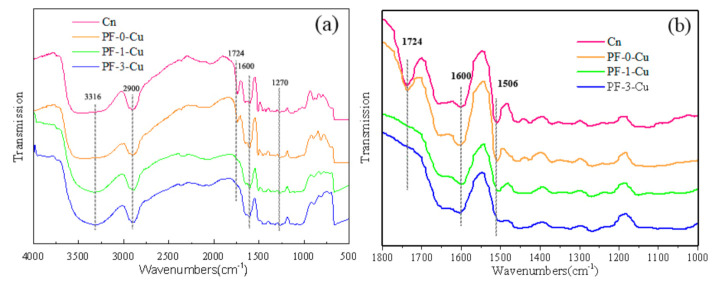
FTIR spectra of the control (**a**), PF-0-Cu, PF-1-Cu, and PF-3-Cu samples (**b**).

The cell walls of Chinese fir are primarily composed of cellulose, hemicellulose, and lignin. It was reported in previous literature that cellulose etherification with formaldehyde or urea-formaldehyde oligomers can react in acidic conditions. This contrasts with the same reaction in neutral or alkaline conditions, where the extents of reactions are low [38,39]. To confirm the possible interactions between the wood cell walls and the modified PF resin in this study, changes in the chemical groups of the samples were detected by FTIR spectroscopy. The FTIR spectra of the samples are presented in Figure 9 and Table 5.

The red and orange curves in Figure 9 denote the control and PF-0-Cu samples, respectively. The absorption at 1724 cm^−1^ of the red curve indicated C=O stretching vibration in the acetyl groups in the hemicellulose [40]. After being impregnated with PF resin (orange spectra), the carbonyl and carboxyl groups’ adsorptions at 1724 cm^−1^ of the control sample shifted to lower wave numbers at 1722 cm^−1^.

Comparison of the PF-0-Cu and PF-1-Cu curves indicate that a chemical reaction occurred among the wood cell walls, nano-copper particles, and PF resin. The C=O stretching vibration at 1724 cm^−1^ disappeared after nano-copper modification, whereas the C–O stretching vibration of aliphatic C–OH, aliphatic C–O, and methylol C–OH at 1270 cm^−1^ were intensified in the modified PF resin curves (green and blue spectra).

From the FTIR spectra of the control samples, PF-0-Cu, PF-1-Cu, and PF-3-Cu, the carbonyl and carboxyl groups in the hemicelluloses of the control sample at 1724 cm^−1^ shifted to 1722 cm^−1^. This finding agrees with reports that PF resin accelerates the oxidation of wood cell walls [41]. This occurrence is consistent with the literature indicating that PF resin and –OH groups in wood polymers can crosslink [18,34]. It is conducive to the adhesive forces across the interphase boundaries between the wood cell walls and PF resin.

Comparison of the curves of the FTIR spectra indicated that the C=O stretching vibration at 1724 cm^−1^ disappeared after nano-copper modification, while the C–O stretching vibration of aliphatic C–OH, aliphatic C–O, and methylol C–OH at 1270 cm^−1^ increased in the modified PF resin curves (green and blue spectra). These changes revealed that the modified PF resin reacted with acetyl groups in the hemicelluloses [42]. The modified PF resin samples showed a relatively larger ratio at 1600 cm^−1^, which was attributed to the enhanced condensation reaction of the methylol in the PF resin in the presence of nano-copper to form methylene (–CH_2_–) [42].

**Figure 10:**
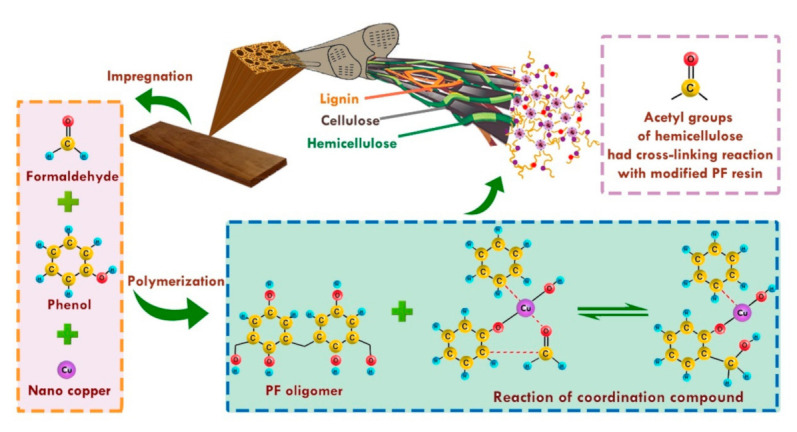
Schematic of the chemical reactions of cell wall components with nano-copper-modified PF resin.

The surface atomic coordination of nano-copper particles is insufficient at high surface energy and chemical activity. Nano-copper particles can easily react with hydroxyl groups in the PF resin to form metal complexes, block the hydroxyl groups, and increase the chemical stability of the PF resin. It has been speculated that the structure of the coordination compound with copper nanoparticles and PF resin may crosslink with the acetyl groups of hemicelluloses [23,43] (Figure 10). Therefore, under the interactions of Chinese fir, PF resin, and nano-copper, the physical properties of Chinese fir were considerably improved. This modified Chinese fir can be utilized in outdoor buildings and furniture.

### 3.5. Novelty, Limitations and Perspectives of the Study

In this study, nano-copper-modified PF resins with good dispersibility were synthesized in situ, with PVP as the surface stabilizer, reducing agent, and dispersant for the uniform dispersion of nano-copper in the PF resins. The bonding strength of the filler-matrix was improved, solving the brittleness problem of the PF resin-treated wood. Consequently, the mechanical properties of Chinese fir were enhanced. In future research, we intend to focus on the weathering test resistance and antibacterial properties of Chinese fir impregnated with nano-copper modified PF resins, which is essential to be utilized in outdoor buildings and furniture.

## 4. Conclusions

In situ synthesized nano-copper modified PF resin is an effective treatment method for Chinese fir, due to the improved diffusive properties and reinforcement of the mechanical properties of Chinese fir. The in situ synthesized nano-copper particles were well-dispersed, and their morphology was basically spherical, with a diameter of about 70 nm in the PF resin. Due to the small size, the nano-copper modified low molecular weight PF resin could penetrate into the S2 layers of the cell walls and interact with them. The modified PF resin crosslinked with the acetyl groups of hemicelluloses of the Chinese fir, and the nano-copper reacted with the hydroxyl groups in the PF resin to form metal complexes, block the hydroxyl groups, and increase the chemical stability of the PF resin. Therefore, the micro-and macro-mechanical properties of the Chinese fir were enhanced after treatment with nano-copper modified PF resin. The PF-1-Cu modified PF resin was identified as the best choice for the modification of Chinese fir, where the average NI MOE and hardness values of the cell walls in the samples increased by 12.8% and 22.2%, respectively, and the impact toughness and compressive strength of them increased obviously 21.8% and 8.2% than that of the pure PF resin, respectively. This study can provide practical guidance for the modification of Chinese fir utilized in outdoor buildings and furniture.

## Figures and Tables

**Table 1:** The main compositions prepared for nano-copper modified phenol-formaldehyde (PF) resin.

Sample	CuSO_4_ 5H_2_O/mol	EDTA-2Na/g	PVP/g	Sodium Pyrophosphate/g	Seignette Salt/g	Deionized Water/mL	Cu/%
Control	-	-	-	-	-	-	-
PF-0-Cu	0	-	-	-	-	-	0
PF-1-Cu	0.05	18.61	0.433	2.659	5.645	25	1
PF-3-Cu	0.15	55.83	1.299	7.977	16.934	25	3

**Table 2:** Viscosity, solids content and pH values of PF resin, alone or amended with nano-copper particles.

Sample	pH	Viscosity (mPa·S)	Solids Contents (%)
PF-0-Cu	10.11	32.6	0.52
PF-1-Cu	11.08	13.9	0.39
PF-3-Cu	11.07	13.8	0.40

**Table 3:** Relative percentage of Cu at each point in Figure 5b.

Sampling Point	1	2	3	4	5	6	7	8	9	10
Cu (%)	2.94	4.11	0	0	2.84	1.41	0	2.04	3.07	1.59

**Table 4:** Changes in cell walls mechanical properties of samples before and after modification treatment.

Samples	Average MOE (GPa)	Average Hardness (MPa)	MOE Increase (%)	Hardness Increase (%)
Control	17.3 ± 0.45 B	415.1 ± 10.92 B	1	1
PF-0-Cu	18.9 ± 0.19 A	491.4 ± 24.58 A	9.9	18.4
PF-1-Cu	19.5 ± 0.48 A	507.3 ± 10.18 A	12.8	22.2
PF-3-Cu	19.6 ± 0.44 A	506.1 ± 10.19 A	13.7	21.9

The MOE, hardness values followed by different letters significantly differ according to the Tukey test (*p* < 0.05).

**Table 5:** FTIR spectrum assignments of the control, PF-0-Cu, PF-1-Cu, and PF-3-Cu samples.

Wavenumbers (cm^−1^)	Assignment
3316	O–H stretching vibration
2900	C–H stretching vibration of methylene
1724	C=O stretching vibration in acetyl groups in the hemicellulose
1600	elongation of C=C
1270	C–O stretching vibration of aliphatic C–OH, aliphatic C–O, and methylol C–OH

## Data Availability

Data sharing is not applicable to this article.
